# Supplementary material for: Are carbon emissions trading and green financial instruments synergistic? -Comprehensive quantitative research based on content analysis
Source: PLoS One. 2024 Mar 7;19(3):e0298601. doi: 10.1371/journal.pone.0298601 (PMC10919601; doi:10.1371/journal.pone.0298601)
Supplement: S1 Data — (ZIP) [file pone.0298601.s001.zip › Data/地级市Z_F工作报告.zip!/╜¡╦╒/│ú╓▌╩╨/└┤╫╘╣1⁄2╓┌║┼í╛┬φ┐╦╩2╛▌═°í┐_2003.docx]

各位代表：

　　现在，我代表常州市人民政府，向大会作工作报告，请予审议，并请市政协各位委员和其他列席人员提出意见。

　　一、工作回顾

　　2002年，是我市全面推进富民强市、率先基本实现现代化的奠基之年，也是常州经济发展速度加快、运行质量提高的丰收之年。一年来，在省委、省政府和市委的领导下，我们以邓小平理论和“三个代表”重要思想为指导，坚持以经济建设为中心，正确处理好改革、发展、稳定的关系，紧紧抓住“三个推进、四个加快”的工作重点，解放思想，与时俱进，真抓实干，奋勇争先，圆满地完成了市第十二届人大五次会议确定的目标任务，经济和社会事业取得了令人鼓舞的成绩，全市呈现出各业兴旺、城乡繁荣、政通人和的喜人景象。

　　——结构调整力度强劲，国民经济实现“四大突破”。在过去的一年里，我们紧紧围绕加快发展这一主题，增加投入，扩大出口，促进消费，强力推进经济结构的战略性调整，国民经济保持了高开高走、快速增长的良好势头。全市国内生产总值完成760亿元，人均国内生产总值突破2万元，达到2.23万元，按现价均增长13%；财政收入突破100亿元，达到102.9亿元，增长35.9%；全市协议利用外资突破25亿美元(原口径)，达到25.7亿美元，增长122%；全社会固定资产投资突破250亿元，增长31.1%，其中工业投入达123.7亿元，相当于“九五”期间总投入的1/3。农业和农村经济在“三化一转移”中增产、增效，农业市场化、产业化、外向化程度明显提高。花卉苗木等特色农业发展步伐加快，龙头企业带动作用日趋明显，“三资”投入开发农业力度加大，粮经比例调整到32：68，农村专业合作组织和农民经纪人队伍发展壮大，小城镇、农田水利等基础设施建设得到加强，乡镇工业产值占全市工业产值的71.8%。现代制造业基地建设全面推进，工业经济发展速度明显加快，运行质量显著提高，传统产业得到改造提升，支柱产业支撑作用增强，高新技术产业发展迅猛，高新技术产品产值占工业总产值的比重达24%。全市工业总产值达到1650亿元，增长16%；规模以上工业利税达到80亿元，增长17.2%。全社会用电量达到99亿千瓦时，其中工业用电量68亿千瓦时，增幅达到26.4%。服务业发展水平不断提高，全年服务业增加值完成280亿元，增长12.3%。全市金融机构贷款余额达560亿元，增长25.8%，各金融单位的资产质量和运行效益均名列全省前茅。商贸流通业态进一步优化，房地产、邮电通信、社区服务等行业发展迅速。旅游业快速提升，荣获“中国优秀旅游城市”称号。同时，各辖市、区在全市经济结构战略性调整和加快发展的大格局中，你追我赶，争先创优，国民经济保持了快速增长的好势头。

　　——体制机制活力倍增，改革开放取得重大进展。坚持把加快改革作为促进经济发展的根本动力。大力推进以产权制度为重点的企业改革，国有集体企业改革转制步伐加快，全年完成市属国有集体企业改制23户，破产、关闭企业25户；黑牡丹、常茂生化成功上市；加大了市场体系建设力度，生产要素市场得到发展和规范；实现了国有企业下岗职工生活保障向失业保险制度的并轨。大力调整所有制结构，私营个体经济发展迅猛，成为我市国民经济的新亮点，私营企业总户数和注册资本分别达到2.4万户和177.2亿元，同比增长34.7%和56.8%，从业人员达到33.4万人，同比增长34.7%。开放型经济以招商引资为重点，以园区建设为突破口，规模和质量跃上新的台阶。利用外资呈现大项目多、增资项目多、高新技术项目多的特点，全年实际利用外资达9.8亿美元(原口径)，增长58.2%。国家级高新区招商引资势头强劲，协议利用外资达到13.5亿美元。省级钟楼经济开发区正式成立，重点乡镇工业小区建设步伐明显加快。完成外贸出口26.6亿美元，增长22.7%。常州高新区海关直通点正式开通，成功地举办了世界中小企业大会和第七届中国国际中小企业商品博览会。

　　——城市建设跨出大步，城乡面貌明显改观。在全市人民和各级干部的共同努力下，顺利实施了市管县体制以来最大的一次行政区划调整，拉开了城市框架，拓展了发展空间，优化了城市布局，更有利于城市资源的有效整合和综合实力的快速提升。确定了建设现代化特大城市的总体思路和目标，明确了三年城市建设的具体任务，初步完成了新一轮城市总体规划纲要的编制。进一步加大城市建设力度，完成交通和市政设施投入42亿元，比上年增长44.8%。常澄、宁杭、锡宜三条高速公路常州段进展顺利，公路大外环的南环线建成通车，怀德路、劳动路等市区主干道得到拓宽，人民公园和兰园敞开，桃园绿地、新城绿地等一批绿化工程相继建成。开展城市管理相对集中行政处罚权试点工作，实施“两级政府、三级管理”的城市管理体制改革，市容环境、交通秩序、清临拆违和马路市场等“四项整治”取得显著成效。积极开展“三城同创”，创建国家卫生城市通过省级考核，国家环保模范城市通过省级调研，荣获“全国创建文明城市工作先进城市”称号，我市和所辖金坛、溧阳两市同时被评为“江苏省文明城市”。经营城市的理念在发展中得以确立，经营城市的实践在规范中不断深入，探索并建立了经营城市的五大运作机制，严格实施土地收储制度，严格实行经营性土地公开招标拍卖。

　　——社会事业蓬勃发展，精神文明建设取得丰硕成果。全面推进学习型城市建设，机关、企业、家庭等六类学习型组织不断涌现，学习日制度、“新三学”活动等十大学习载体建设富有成效，为全市干部群众整体素质的提高打下深远而坚实的基础。科技作为第一生产力的作用更加明显，科技进步对工业和农业增长的贡献份额分别达到43%和56%，又一次被评为全国科技进步先进城市，并被确定为全国制造业信息化工程重点城市。教育结构进一步优化，新批准建立高等院校7所，80%的职校成为部、省重点学校，各乡镇全面达到教育基本现代化标准。高质量普及12年基础教育。高考万人本科进线率连续第五年居全省第一，基本实现高等教育大众化。按照“政府引导、学校主体、统一规划、市场推动”的运作机制，启动了国内首家以高等职业教育为显著特色的大学城建设，有力地推动了科教兴市战略的实施，有力地促进了武进新区的建设。文化、广电事业迅速发展，群众艺术馆等一批文化设施建成投运，一批作品荣获国家级大奖。卫生事业现代化水平不断提高，市一院病房楼、120急救指挥系统等相继建成，中、西医重点专科建设在全省名列前茅。竞技体育取得优异成绩，群众体育全面发展，第六次被评为全国全民健身活动先进城市。城乡计划生育管理体制改革得到深化，全市人口自然增长率控制在0.2‰以内。群众性精神文明创建活动成果丰硕，民主法制建设不断加强。依法打击各种违法犯罪活动，有效地维护了经济秩序和社会稳定，被评为全省社会治安综合治理先进市。国防动员、国防教育、双拥共建和民兵预备役工作扎实有效，预备役通信团全面建设有了长足进展。新闻出版、地方志、档案、民族宗教等各项工作都取得了新的成绩。

　　——就业保障稳步推进，人民生活水平跃上新的台阶。高度重视和大力加强就业与再就业工作，全市完成城镇就业2.5万人，城镇登记失业率为3.42%。实现国有企业下岗职工基本生活费和企业离退休人员基本养老金按时足额发放。城镇职工医疗保险覆盖面达85%，提高了32.8个百分点，解决了4700多名破产、关闭企业职工的医保问题。切实关心低收入人群的生活困难，深入开展访贫问苦送温暖活动，积极扩大城市低保覆盖面，做到了“应保尽保”。城乡人民医食住行条件明显改善，生活水平有了新的提升。2002年城镇居民人均可支配收入(原口径)、农民人均纯收入分别突破1万元和5000元，达到10759元和5138元；群众受益面较大的社区建设、菜市场改造、危旧房改建等工作进展顺利，为民办实事项目全部完成；固定电话和移动电话用户双双突破百万户；城市居民人均住房使用面积达17.44平方米；民政、残联、信访、老龄、妇女儿童、关心下一代等方面的工作水平有了新的提高。

　　——政府职能加快转变，投资创业环境日趋优化。按照WTO规则和建立社会主义市场经济体制的要求，以转变政府职能、提高行政效能为中心，大力推进政府自身建设与改革。第二轮行政审批制度改革取得新的成果，审批总事项由927项减少到462项，减幅达50.2%，并率先在全省实行企业登记注册并联审批。切实加强政府工作的规范化和制度化建设，深入开展政务公开，建立行政效能监察机制，行政效率和服务水平明显提升，基层企业和人民群众的满意程度不断提高。深入推进反腐倡廉，进一步完善廉政建设责任制，认真纠正行业不正之风。着力加强增收节支工作，规范政府采购行为，6个部门的部门预算试点工作成效显著，涉及264个单位的部门预算编制工作全面推开。大力整顿市场经济秩序，加大打假治劣、安全生产、公平交易以及建筑、文化、运输等市场的专项整治力度，推进创建金融安全区活动，市场秩序和经济环境得到较大改善。推行涉农收费公示制度，农村税费体制改革及配套措施进一步深化，有效地减轻了农民负担。同时，各级政府自觉接受同级人大及其常委会的监督，重视和发挥政协以及各民主党派、工商联、人民团体的政治协商和民主监督作用，人大代表、政协委员提出的议案、建议和提案已全部办结。

　　各位代表，在全市人民的共同努力下，本届政府全面完成了市第十二届人大各次会议确定的目标任务。过去五年，是我市综合实力提升最快、改革开放力度最强、城乡面貌变化最大、人民群众得益最多的时期。五年来，全市国内生产总值年均增长11.3%；财政收入年均增长20.7%，其中地方财政收入年均增长23.4%；固定资产投资累计达到889亿元，为前五年的1.6倍；三次产业的比重由1997年的9.4：56.6：34调整为2002年的6.5：56.7：36.8。五年来，全市实际利用外资累计达31.7亿美元，比前五年增长145%；外贸出口累计达92.69亿美元，年均增速16.6%；以国家级高新区为龙头的三级开发园区快速发展，已成为我市新的经济增长区域，促进了全市生产力布局的优化调整。五年来，实施改革转制的市属国有集体企业达218家，累计上市企业7家，乡镇企业改制任务基本完成，非公有制经济在国民经济中的比重大幅提升，多种经济成份实现了共同发展。五年来，全市城市交通、市政、供电、电信等基础设施累计投入达217.5亿元，市区人均公共绿地达6平方米，城市化率由40%提高到55%。五年来，全市城镇居民人均可支配收入和农民人均纯收入年均增速分别达到9.4%和4%，居民储蓄存款余额达到500亿元，人均预期寿命达76.3岁，人民群众的居住条件、生活环境、医疗卫生和文化生活条件有了明显改善。

　　回顾过去，全市人民在共同奔向现代化的征程中取得了令人鼓舞的巨大成就，在富民强市的实践中焕发出催人奋进的精神力量。五年来取得的成绩来之不易，五年来积累的经验弥足珍贵。展望未来，我们恰逢中华民族伟大复兴的大好时光，我们正面临着常州凝心聚力、蓄势腾飞的大好时机。在这承前启后、继往开来的时刻，对全市人民在改革、开放、发展中所创造和积累的成功经验，应该认真总结、发扬光大。归结起来主要是：

　　必须始终坚持与时俱进、开拓创新的思想品格。面对科学技术日新月异、突飞猛进的发展态势，面对方兴未艾的全球经济结构调整和产业重组，面对不断深化的国内经济体制转轨和社会转型，我们只有勇于探索、敢于攻坚、善于创新，以创新的思维、创新的胆略、创新的举措，冲破一切妨碍发展的思想观念，改变一切束缚发展的做法、规定，革除一切影响发展的体制弊端，才能谋划高人一筹的新招实招，取得快人一拍的业绩实绩。

　　必须始终保持抢抓机遇、奋力拼搏的精神状态。机遇是极其宝贵的发展资源。机遇属于有准备、勤于积累的人们，属于有眼光、审时度势的人们，属于有胆识、敢为人先的人们。我们只有牢固确立率先意识、机遇意识、拼搏意识和责任意识，才能紧紧抓住大改革、大开放、大调整、大转折带来的新机遇，不断推进改革开放和国民经济持续、快速、高效发展。

　　必须始终确立争先抢位、勇攀高峰的发展目标。在不进则退、慢进也是退的竞争态势中，在先进地区快速发展和相对落后地区全力赶超的双重压力下，我们只有不断鼓劲加压，拉高起点，突出重点，全面推进，才能不断地追求一流、追求超越，积蓄更大的发展后劲，拓展更大的发展空间，形成空前强大的凝聚力和战斗力。

　　必须始终弘扬脚踏实地、勇于实践的工作作风。落实富民强市的大政方针，完成改革、发展、稳定的艰巨任务，关键靠苦干、实干。我们只有立足实际，注重实效，坚持快节奏、高效率，在抓落实上下真功夫、硬功夫，才能切实化解各种矛盾，及时克服各种困难，确保各项事业不断取得新的胜利。

　　必须始终把握执政为民、以人为本的服务宗旨。任何时候都要以富民的目标凝聚人心，以富民的举措激励人心，以富民的业绩鼓舞人心。我们只有顺应民意，造福于民，充分尊重劳动、尊重知识、尊重人才、尊重创造，让想干事的有机会、能干事的有舞台、干成事的有地位，才能使一切创造社会财富的源泉充分涌流，才能激发全社会方方面面的积极性，把我市的社会主义现代化建设事业不断推向新的高潮。

　　各位代表，过去五年所取得的辉煌成就，是全市人民在中共常州市委的领导下，团结奋斗、锐意进取的结果，是人大、政协监督支持的结果。在此，我代表市人民政府，向积极投身于改革开放和现代化建设事业的全市人民，向给予政府工作热情支持与监督的人大代表、政协委员、各民主党派、无党派人士、工商联和各界人士，向驻常部队指战员和武警官兵，向所有关心、支持常州建设和发展的海内外朋友们，表示衷心的感谢!

　　在充分肯定成绩的同时，必须清醒地看到，我市经济和社会生活中还存在不少困难和问题。主要是：经济的结构性矛盾仍较突出，推动经济快速发展的大企业、大项目偏少；经济发展的体制性障碍尚未消除，企业改革转制必须加大攻坚力度，国有资产管理体系有待进一步健全；城市化推进过程中长效管理机制和经营机制亟待完善；市本级财政收支矛盾突出，增收节支的任务艰巨；农民增收、城镇就业和社会保障压力较大，影响社会稳定的因素依然存在。同时，机关作风建设和公务员队伍建设需要进一步加强。对此，我们要高度重视，在今后的工作中认真加以解决。

　　二、主要工作任务

　　二十一世纪头二十年，是我们必须紧紧抓住并且可以大有作为的重要战略机遇期。今后二十年，我国要集中力量，全面建设小康社会。对我们常州来说，要在全面建设小康社会基础上，率先实现现代化。实现这一宏伟目标，今后五年至关重要。我们要按照市第九次党代会确定的各项任务，促进社会主义市场经济运行机制更趋完善，基本形成特大城市发展格局，实现经济综合实力和整体素质再上新台阶，确保国内生产总值和财政收入实现翻一番，不断提高人民生活富裕水平和社会文明程度，努力使城乡人民收入持续增长、生活更加富足。

　　今年是贯彻党的十六大精神、开启新征程的第一年，也是新一届政府任期的第一年。做好今年的工作，具有十分重要的意义。展望今年的形势，世界经济复苏前景看好，我国经济运行的国际环境将更加好转；党的十六大为经济发展注入了强劲动力，国内经济体制和市场环境将进一步变革和优化；国际资本和产业大举向长江三角洲转移，必将给我们带来新的经济增长空间；上海“申博”成功，为地处上海经济圈的我市带来了新的商机。同时也应看到，国际政治经济形势复杂多变，贸易保护主义有所抬头，十分激烈的国内竞争又面临国际强手的挑战，周边城市之间的抢先争位更加激烈。我们既要看到国内外经济形势提供了加快发展的有利条件，又要看到面临的不利因素，更要看到我市改革开放和现代化建设中蕴藏着巨大的增长潜力。总之，今年是充满希望和面临考验的一年，是机遇多于挑战，希望大于困难的一年。我们一定要增强加快发展的信心和决心，正视竞争和发展中的现实差距和压力，抓住市场大开放、产业大调整带来的难得机遇，奋力开拓，锐意进取，再掀各项事业发展的新高潮。

　　2003年政府工作的总体要求是：以邓小平理论和“三个代表”重要思想为指导，全面贯彻党的十六大精神，紧紧围绕富民强市、率先基本实现现代化的总目标，高扬发展主题，突出调整主线，开放开发构筑更高平台，体制机制创造更强活力，城建城管展示更新面貌，农村农业实现更快提升。强力推进三级开发园区建设，实现利用外资、民资再上大台阶；强力推进市场取向改革，全面完成国有集体企业改革转制任务；强力推进现代化特大城市建设，进一步加大管理和投入力度；强力推进县区经济快速发展，加快富民强市步伐，努力实现经济持续快速健康发展和社会全面进步，夺取全市改革开放和现代化建设的新胜利。

　　2003年国民经济和社会发展的主要预期目标：国内生产总值增长12%，财政收入增长15%，全社会固定资产投资增长30%，外贸出口增长15%，协议利用外资增长50%，实际利用外资增长30%，社会消费品零售总额增长12%，城镇居民人均可支配收入增长10%，农民人均纯收入增长6%，城镇登记失业率控制在4%以内，人口自然增长率控制在1‰以内。

　　为实现上述各项目标任务，今年要着重抓好以下七个方面的工作：

　　(一)着力加快各类开发园区建设，推动开放型经济再上新台阶

　　坚持把开放型经济作为带动全市经济快速发展的重要动力，进一步形成全市上下狠抓开放型经济的强大合力，以更高的目标，更有力的措施，更优越的环境，在更大范围、更广领域和更高层次上参与国际经济技术合作与竞争，确保开放型经济增长快于国内生产总值增长，高于全省平均水平。

　　强力推进开发园区建设，在规模总量上求得新突破。把各级各类开发园区建设作为今年开放型经济工作的重中之重，充分发挥其对外开放的窗口、经济发展的龙头、辐射带动的基地、体制创新的先行和城镇化推进的示范作用。充分挖掘国家级高新区的强力带动作用，积极推进新一轮创业特别是滨江工业园区的整体开发，加快电子产业园、软件园、环保园等专题园区建设；引导和支持省级开发区加快基础设施建设，着力提高承载功能，办好“区中园”，争创新优势；因地制宜、统筹布局，高起点规划和建设乡镇工业小区，培育发展一批集约式、环保型、特色化的重点乡镇工业小区。同时，积极改革和创新开发园区的管理体制和运行机制，形成与国际接轨的管理方式和创业环境。今年，各类开发园区利用外资的总量要占到全市的80%以上，各开发区、重点乡镇工业小区的基础设施投入和外资民资的投入均增长50%以上。

　　加大招商引资力度，在利用外资上再攀新高峰。继续把台湾、香港地区和日本、韩国作为招商引资的重点区域，组织好赴日、韩大型招商活动，主动出击，全力强攻，力争取得重大突破。突出抓好电子信息、生物医药、新型材料等高新技术产业以及重化工、环保产业等项目的招商引资，力争形成更为先进的产业结构和关联集中的企业集群。不断拓宽利用外资领域，提升外资在农业、社会事业、基础设施和服务领域的份额。完善重大项目的快速反应、综合协调和跟踪服务机制，大力开展区局联手招商，吸引国际知名企业及其研发机构落户常州，努力实现重大引资项目的新突破。年内力争新批超千万美元项目100个，其中超亿美元项目3-5个。注重签约项目的实际缴资率和开工、竣工的考核，不断提高招商引资的成效。同时，进一步扩大对内开放，主动接受上海经济辐射，积极参与“世博经济”，加强与兄弟城市及国内大集团的横向联合和协作配套。

　　全方位拓展国际市场，在对外经贸上创出新水平。大力实施市场多元化战略，调整优化出口商品结构，努力扩大有比较优势的机电、纺织服装及高新技术产品的出口，今年外贸出口力争突破30亿美元。推进外贸经营主体多元化和贸易方式多样化，扩大服务贸易和加工贸易，新增有外贸经营权的企业200家，出口创汇超1000万美元的企业40家。积极实施“走出去”战略，引导有比较优势的行业及企业到国外投资办厂，努力扩大技术型、管理型劳务输出，确保今年外经营业额增长10%。加快建设出口加工区，加强税贸、关贸、银贸协作及国检、口岸、外汇管理等相关部门的协调配合，提高通关效率。引导和支持企业开展国际标准、安全和环保认证，使更多的企业和产品获得进入国际市场的“通行证”。

　　(二)着力加快工业结构战略性调整，全面推进现代制造业基地建设

　　加快实施现代制造业基地发展纲要，围绕三大产业群、七个专业化制造基地建设，狠抓工业结构调整优化和产业升级，走新型工业化道路，进一步发挥工业对经济社会发展的支撑和推动作用。今年规模以上工业增加值增长13%以上。

　　强化科技创新，促进产业升级。积极实施以企业为主体的科技创新工程，年内新增4家省级以上企业工程研究中心、技术开发中心和博士后工作站，实施50项重点科技和产学研项目，新增省级以上高新技术企业10家。完善科技创新的政策环境和风险投资机制，加快科技服务体系建设，办好创业中心、留学生创业园等创新载体。坚持以信息化带动工业化，充分利用我市被确定为“全国制造业信息化工程重点城市”的有利条件，大力运用信息技术提升传统产业的信息化水平。抢抓国际信息产业梯度转移的有利时机，在数字视听、软件、计算机及网络、新型电子元器件、通信装备及机电一体化产品的研发、生产上，形成自身特色和产业链优势。同时，积极发展环保、新型材料、生物医药和精细化工等新兴产业，使之尽快成为新的经济增长点。

　　加大有效投入，迅速增强工业发展后劲。把加强工业技术改造和重大项目建设放到突出位置，大力营造全市上下齐心协力抓投入、上项目、增后劲的浓厚氛围。进一步拓宽投资渠道，大中小项目并举，外资、民资、金融资本齐上，努力实现投入总量、项目规模的新突破。继续开展工业投入“六比六赛”活动，鼓励各类企业围绕市场需求，扩大增量投资。积极争取国家“双高一优”和国债专项计划，落实技改项目贴息，扩大信贷规模，保证重点项目顺利实施。今年确保完成技改投入160亿元，增长30%以上。同时，加快在建项目的进度，抓好已建项目的投产达标，建设好重大项目储备库。

　　坚持扶优扶强，积极实施大企业大集团战略。把培育大企业大集团，作为提升我市现代制造业水平的重要举措，发展一批主业突出、拥有自主知识产权、核心竞争力强的大型企业集团。引导重点企业通过资金注入、技术转让、管理输出等途径，大力推进跨地区、跨行业、跨所有制的并购重组。在政府引导、企业自愿的原则下，搞好强强联合，集聚优质资产，壮大经营规模，实现快速裂变扩张。切实加强重点企业的品牌建设，以品牌为龙头整合资源，拓展市场。引导已上市企业规范运作，加快重点企业上市步伐，力争年内有1-2家企业上市。今年争取年销售收入超20亿元的企业7-8家，其中超过50亿元的企业2家。与此同时，积极扶持发展面广量大的中小企业。

　　(三)着力加快致富农民步伐，促进县区经济实现新跨越

　　全面奔小康，重点在农村。按照中央关于“更多关注农村、关心农民、支持农业”的要求，大力发展现代农业，加快县区经济发展，千方百计增加农民收入，构筑加快农村现代化步伐的物质基础。

　　大力推进农业产业化经营，切实提高市场化组织程度。继续实施龙头带动战略，发展壮大江苏晨风等一批国家、省、市级农业龙头企业。发挥我市农业资源丰富、平原圩区与丘陵山区兼备的比较优势，加快建设花卉苗木、特种水产、食草畜禽等产业优势突出的农产品生产基地。积极引导民间资本、工商资本和外资发展农副产品加工业，完善各类农副产品批发交易市场，努力构建贸工农、产加销一体化经营体系。大力发展互助互惠的各类专业合作组织、农产品营销中介机构及农民经纪人队伍，建立健全功能完备、手段先进的农业服务组织。积极发展都市农业、生态农业和观光旅游农业，使之成为新的农业增长点。

　　大力开展农业标准化建设，改善农村生态环境和基础设施条件。加强农村工业“三废”和农业面源污染治理，切实提高农业生产环境和农村生态环境质量，从源头把好农产品质量关。建立健全农产品质量标准和检验检测体系，大力推进农产品市场准入制度。认真抓好农业标准化示范园区建设，积极发展无公害食品、绿色食品和有机食品，力争今年再建成40万亩无公害农产品生产基地。切实抓好武宜运河拓浚、北河拓浚三期等重点水利工程建设，加强河道清淤、圩堤加固、灌区改造、乡镇供水等农业和农村基础设施建设，继续推进土地复垦开发整理和农田林网建设。把小城镇建设同发展乡镇工业小区、完善农村市场体系、发展农村服务业结合起来，加快我市农村城镇化、现代化进程。

　　大力拓展非农产业，加快农村劳动力转移。加快农村工业化步伐，进一步发挥村镇工业就地吸纳农村劳动力的主渠道作用。大力发展劳动密集型的农副产品加工业，促进农村劳动力内部消化。进一步放大我市“建筑之乡”、“花卉之乡”的优势，积极发展建筑业、园林业，全力开拓国内外市场，结合资本、技术、管理对外输出，促进农村劳动力异地有序转移。引导和规范农户土地承包经营权流转，取消对农民进城经商务工的不合理限制和收费，促进农村人口向城镇和非农产业转移。提高劳务输出中介服务水平，有计划、有针对性地开展技能培训，切实提高农村劳动力就业能力。

　　(四)着力加快体制机制创新，增添经济社会发展新动力

　　体制机制的变革，是经济发展的强大动力。围绕今年全面完成国有、集体企业改革转制的目标，重点抓好产权制度改革和完善法人治理结构两大关键环节，着力推进市场取向改革向多领域、多行业拓展，努力形成各行各业齐抓改革、锐意创新的新局面。

　　加快国有经济战略性调整，推进国有企业改革转制。加快国有资本退出步伐，全面完成市属国有、集体企业的改革转制任务，采取经营者入股、引进外来资本、资产转让等方式，实施投资主体多元化；突出抓好“大而盈”、“大而亏”、“大而难”企业的改制，妥善解决人员分流、身份置换、资产调整、土地处置、债务处理等问题；全面退出中小企业中的国有资本，放开股权受让对象，放开股权出让方式，大力推进中小企业民营化；关闭淘汰已经停产、资不抵债的国有、集体企业，有序推进劣势企业退出市场。对于已经改制的大中型企业，要通过建立规范的法人治理结构，形成责权统一、协调运转、有效制衡的运作机制。深化国有资产管理体制改革，建立健全权利、义务和责任相统一，管资产和管人、管事相结合的国有资产管理体制。

　　创造宽松环境，充分释放私营个体经济的巨大潜力。着力优化私营个体经济发展环境，促进总量上规模、管理上水平、实力上台阶，使之成为我市最具活力的富民经济，今年私营个体经济增加值增长20%以上。进一步拓展私营个体经济的发展空间和领域，鼓励私营个体企业参与国有企业改组改造和资产重组，以独资、合作、联营参股等方式参加经营性基础设施项目建设，投资科教文卫等领域；进一步落实发展私营个体经济的政策措施，建立健全中小企业信用担保体系，加大对私营个体经济的金融信贷支持和服务力度；进一步促进私营个体经济裂变式发展，重点扶持一批规模型、科技型、外向型民营企业，重点建设一批具有一定规模的私营经济园区，重点培育一批技术含量高、市场前景好、附加值高的名牌产品，提高私营个体企业的竞争能力。

　　坚持市场取向，积极推进社会事业改革。按照政事分开的原则，坚持社会事业公益化与市场化、产业化相结合的发展方向，改革事业单位的用人、分配制度，实施产权制度和机构改革，尽快完成第一批市属生产经营型事业单位的改制转企任务。深化医保、医疗、医药联动改革。加强住房公积金管理，实施区级房管体制改革，加快住房分配货币化步伐。与此同时，进一步巩固和深化农村税费及其各项配套改革，继续推进粮食购销市场化。

　　加快改革与发展，增强服务业对经济增长的拉动力。把加快发展服务业作为经济结构调整的着力点，不断提升发展水平。今年全市服务业增加值占国内生产总值的比重达到37.1%，从业人员占全社会的比重达到29%。拓展创新金融保险业，吸引国内外银行和保险公司到常州设立分支机构，鼓励有一定实力的单位和个人投资金融领域；大力发展金融衍生业务，不断完善个人消费信贷体系。培育具有现代经营理念、现代经营业态和现代经营环境的商业物流产业，推动综合物流园区和物流中心建设。大力发展旅游业，打造“中华龙城”旅游品牌，加快淹城、太湖湾旅游度假区和茅山旅游度假区等一批重点旅游区(点)建设，积极推进天宁宝塔、天目湖“动画城”、龙虎园等项目，尽快形成新的旅游热点；着力推进旅游业的改革转制和联合重组，培育龙头旅游企业。高起点发展信息服务业，建设“数字常州”。全面完成商贸流通、粮食、供销等行业的国有、集体企业改革。完善市场体系建设，加快专业市场改革步伐，并认真做好第二次三产普查工作。

　　(五)着力加快城市现代化进程，促进城市管理和投入再上新水平

　　今年是实施城市建设三年纲要的第一年。我们要以更大的手笔、更大的气魄、更大的力度，狠抓规划、建设、管理三大环节，积极实施构筑区位优势、增强城市功能、营造城市环境和美化城市形象等四大系列工程，进一步完善科学高效的城市规划管理、多元化的城建资金筹集、市场取向的市政公用行业运行、严格长效的城市综合管理和规范严格的工程投资监管等五大机制，努力塑造城市新形象，加速推进城市化和城市现代化。

　　强化基础工作，实施长效管理。按照“注重基础、强化整治、立足长效、全面提高”的要求，坚持规划的龙头地位，强化规划的集中统一管理；完成城市总体规划修编工作并上报审批，编制好一批专项规划和重点地区的控制性详细规划。坚持规划管理的科学化、民主化，积极实行规划的公示、展示、听证制度，提高规划的透明度和决策水平。全面推行城市管理相对集中行政处罚权工作。积极实施以块为主、分级管理的“两级政府、三级管理”体制，形成市和区联动、部门与街道社区共抓、覆盖全市的城市管理网络体系。深入开展“三城同创”活动，年内确保国家卫生城市和国家环保模范城市创建成功，为创建全国文明城市打下坚实基础。继续开展城市环境四项综合整治活动，建立巡查制度，重点加强对市容卫生、违章建筑、城市出入口、城乡结合部和机动车特别是摩托车的治理，使城市形象得到更为明显的改观。与此同时，进一步加强社区建设与管理，深入开展新型小城镇创建活动。

　　创新城建投融资机制，全面提高经营城市水平。建立健全城市建设投入、回报、偿还、再投入的良性循环机制。进一步加大城市建设招商引资力度，举办好城建招商会。强化、完善土地收购储备制度，严格实行经营性项目土地使用权公开挂牌、招标、拍卖出让，提高净地出让率，城市土地收益实现新的突破。全面实行公益性城建项目公开招投标和全程审计，强化内部管理，完善外部监督，提高工程质量和投资效益。加快环卫、绿化、公交等市政公用行业的改革，打破行业垄断和独家经营，努力实现投资多元化、运营市场化、发展产业化。

　　加大投入规模，再掀建设高潮。全年计划完成交通、城建等基础设施建设投入80亿元，比上年增长90%。在大交通建设上，加快推进锡宜、常澄、宁杭高速公路建设，启动建设常宁、扬溧高速公路常州段，其中锡宜高速公路和宁杭高速公路先导段建成通车；全面建成常州公路大外环，开工建设金武线、苏239线等一批干线公路，实施锡溧漕河整治工程。在城市道路建设上，重点建设和拓宽西环一路、西环二路、青洋路、兰陵路、清凉路等五条干道，畅通南北交通；继续完善城市路网，拓宽改造勤业西路、东方大道、采菱路、河海西路、武宜南路等市区道路。在市政公用设施建设上，重点建设天然气一期工程，新建郑陆、吕墅等输变电工程，完成常武地区供水、戚墅堰污水处理和夹山生活垃圾填埋场改扩建等工程，同时加快建设新港污水处理厂。在旧城改造和建设上，完成火车站广场综合整治工程，推进南大街商业步行街、西瀛里地块和延陵东路北侧等大规模旧城改造工程，完成人防地下工程5万平方米。进一步做好江边电厂、伍员山蓄能电站、西绕城高速公路、京杭运河市区段改线、常泰过江通道工程等项目的前期工作。与此同时，推进金坛、溧阳县域中心城和重点镇、中心村建设，使之与常州特大城市的建设相配套、相协调，形成合理布局、众星拱月、相互衔接、功能互补的现代化城镇体系新格局。

　　(六)着力加快就业和社保体系建设，千方百计解决困难群众的生产生活

　　就业是民生之本、民安之基。把促进就业、加强社会保障作为事关全局的重要工作来抓，加大就业与再就业工作力度，创新社会保障运行机制，完善社会救助制度，努力维护和保障群众的根本利益。

　　切实做好就业与再就业工作。积极落实有关鼓励用工政策，开发更多的就业岗位。大力发展就业容量大、劳动力需求多的产业和行业，努力拓展就业空间。把深化国有企事业单位改革与积极妥善地安置好职工有机结合起来，落实促进下岗失业人员再就业的有关扶持政策。引导下岗职工和失业人员转变择业观念，鼓励他们自谋职业和自主创业。继续开展再就业援助活动，重点做好“4050”人员的再就业帮扶工作。强化再就业培训，加快劳动力市场建设，逐步形成劳动者自主就业、市场调节就业、政府促进就业的新机制。

　　不断增强社会保障功能。努力扩大社会保险覆盖面，对社会保险费依法实行强制征收，确保征缴率达到95%以上。完善和加强社会保险费的管理，增强社会保险基金的统筹、保障能力，确保医疗、工伤、失业等保险基金年度不出现赤字。做好部省属企业属地参保工作，完善基本医疗保险制度，确保人民群众享有基本医疗。加快推行农村养老、医疗保险，积极发展补充社会保险和个人储蓄性保险，满足多层次、多方面保障需求。切实做好“两个确保”、“低保”和“三条保障线”的衔接工作。完善街道、社区劳动保障网络，健全社会保障服务体系，为群众提供更加便捷、周到的服务。

　　大力加强社会救助和帮困工作。以城乡居民最低生活保障为基本内容，不断完善社会救助制度和运作机制，高度关注社会困难群体，切实保障困难群众的基本生活。开展对困难家庭的普查，确保社会救助对象不遗漏、不重复。扩大城乡“低保”覆盖范围，着力解决好重点优抚对象和特困家庭的实际困难。加强社会福利、老龄和残疾人事业，积极发挥困难职工援助中心的作用。大力发展社会慈善事业，开展社会互助救济、帮困扶贫，逐步完善贫有所济、难有所帮、病有所医、老有所养的社会救助保障体系。

　　(七)着力加快社会事业繁荣发展，促进社会全面进步和人的全面发展

　　坚持物质文明、精神文明与政治文明的共同进步，坚持经济、社会与环境的协调发展，按照建设现代化特大城市的要求，完善社会事业发展规划，为改革开放和经济建设提供强有力的思想保证、智力支持和精神动力。

　　深入推进学习型城市建设，提高市民综合素质和城市竞争力。以六类学习型组织、十大学习载体为抓手，进一步完善行政推动和全民自觉参与相结合的工作体系，积极构建广覆盖、多层次、开放式的全民学习教育体系。继续深化学外语、学电脑、学科技知识的“新三学”活动，着力营造浓厚的学习氛围。建立并完善建设学习型城市的促进机制、动力机制和激励机制，加强学习型城市理论研究，以创新的理论指导创建学习型城市的实践，促进学习型城市建设走上经常化、制度化、规范化轨道，不断丰富创建文明城市的内涵。

　　全面提升教育水平，加快人才资源开发。把教育放在优先发展的战略地位，扎实推进素质教育，实施区域教育现代化工程。加快中小学教育信息化建设，发展网络远程教育。提高高等教育大众化水平，适龄青年进入高校的比例有新的提升。加快职业教育特别是高等职业教育的改革与发展，按照建设精品之作、经典之作的标准，加快推进“大学城”建设，年内完成建筑面积65万平方米，确保1.65万名学生入城就读；经过几年的努力，使之成为投入达25亿元、在校生达10万人，在全省乃至全国一流的高等职业教育基地。积极发展社区教育和职工教育培训，逐步建立终身教育体系。鼓励社会力量办学，发展民办教育。加快高层次人才和国外智力引进，完善招才、育才、用才的人才政策和服务体系，建立人才竞争、激励、评价、分配机制，启动实施机电信息等六大人才高峰行动，继续抓好省“333二期工程”和市“831”学术带头人培养工程，积极构筑新世纪常州人才高地。

　　创新体制机制，协调发展文化卫生事业。把发展文化事业和文化产业作为增强城市综合竞争力的重要组成部分。着力建设历史文化名城，努力提升城市文化品位。积极推进藤花旧馆、意园的修复工程，启动博物馆和“阳湖遗风”规划的前期工作。弘扬主旋律，提倡多样化，繁荣文艺创作，发展广电事业，规范文化市场管理，重视发展哲学社会科学事业，为人民群众提供更多、更健康的精神食粮。加快卫生事业的现代化进程，推进市一院门急诊大楼和市中医院、妇幼保健医院病房大楼等重点项目建设。强化疾病预防控制和卫生监督，加强社区卫生服务和农村卫生初保体系建设，加快非基本医疗和特需医疗保健服务的市场化、产业化步伐。推进计划生育综合改革和优质服务，稳定低生育水平。积极开展全民健身活动，提高竞技体育水平，办好第十二届市运动会。与此同时，认真做好民政、接待、信访、档案、外事、侨务、人防、民族宗教、对台事务、民兵预备役、关心下一代等工作，积极发展妇女儿童事业。

　　坚持依法治市和以德治市，提升城市文明形象。认真贯彻《公民道德建设实施纲要》，加强社会公德、职业道德和家庭美德教育，深入开展爱国主义、集体主义、社会主义思想教育，提升市民现代文明素质，塑造健康向上的人文环境。认真开展双拥活动，实现全国双拥模范城“三连冠”。认真实施“四五”普法教育，切实提高全体市民特别是各级干部的法制观念和依法办事能力。充分尊重人民代表管理国家和社会事务的权利，发挥人民政协以及各民主党派、工商联、人民团体、无党派爱国人士在政治协商、民主监督和参政议政方面的作用。扩大基层民主，进一步推行和规范厂务公开、村务公开。开展诚实守信、依法经营教育活动，建立完善企业和个人的信用档案，健全对失信企业和个人的惩罚机制，营造良好的信用环境。严厉打击各种违法犯罪活动，坚决扫除社会丑恶现象。坚持打防结合、预防为主，落实社会治安综合治理的各项措施，营造更加安全、安定、安宁的社会环境。

　　三、建设高效服务型政府

　　建设勤政廉政、求真务实的人民政府，树立充满生机和活力的政府形象，不断提高政府工作的能力和效率，是加快常州经济社会发展、在全面建设小康社会基础上率先基本实现现代化的迫切需要。我们要紧紧围绕提高行政水平和服务质量，以迅速提升环境竞争力为主线，以推进行政效能建设为着力点，进一步转变政府职能，改进工作作风，建立起行为规范、运转协调、公正透明、廉洁高效的行政管理体制。

　　深化行政管理体制改革，加快转变政府职能。以建设高效服务型政府为目标，构筑新型的政府、企业、市场关系，不断提高驾驭市场经济的能力，切实把政府职能转变到经济调节、市场监管、社会管理和公共服务上来。继续推进行政审批制度改革，切实减少和依法规范行政审批，积极推行并联审批和网上审批，简化环节，缩短时限。积极培育和发展各类行业协会和中介组织，逐步形成规范有序的中介服务市场，充分发挥其服务、自律、代理、协调等功能。加快建立公共财政框架，全面推行部门预算，进一步强化预算外资金的使用和管理，开展国库集中支付试点，狠抓增收节支工作。全面、深入、规范地推行政务公开，对关系人民群众切身利益的重大决策事项实行听证制度，提高政府运作的透明度。

　　切实加强行政效能建设，不断优化发展环境。努力营造廉洁高效的政务环境、规范有序的市场环境和富民安民的生活环境。自觉接受社会和群众的监督，组织开展政府机关“让人民满意”公开评议活动。积极创建学习型机关，提升公务员队伍的整体素质，人人争做勤奋学习、善于思考的模范，解放思想、与时俱进的模范，勇于实践、锐意创新的模范。切实改进领导方式和工作作风，大力精简会议和文件，深入基层开展调查研究，体察民情，吸纳民意，增强广大干部执政为民的公仆意识。强化行政执法监督，规范执法行为，实施行政过错责任追究，发挥市行政效能监察和投诉中心的作用。进一步加强廉政建设，切实抓好反腐倡廉，坚决纠正部门和行业不正之风，努力从源头上、机制上、制度上预防和治理腐败。进一步完善政府采购、建设项目招投标、土地使用权出让等制度，培育发展公平、公正、透明、高效的产权交易市场。继续整顿和规范市场经济秩序，严厉打击制假售假、行骗欺诈等不法行为，依法保护投资者、生产者和消费者的合法权益。

　　着力为民办好实事，不断提高群众生活质量。今年着力为民办好10件实事：(1)新增就业岗位4万个，促进2.5万名城镇人员就业，完成各类再就业培训2.1万人次；(2)改善群众居住条件，改造城区危旧房1500户，解决“双特困”家庭廉租房150套，整治丽华二村等9个住宅小区共200万平方米，发放住房公积金贷款3.5亿元；(3)新增城市绿地200公顷，其中公共绿地100公顷，建成园艺博览园、怀德广场、青山广场、东方广场、新城绿地二期、北港绿地一期、五星绿地、红东绿地、南市河西瀛里段滨河绿地，敞开芦墅公园，开工建设南田公园、淹城森林公园；(4)优先发展公共交通，新购公交车140辆，并解决机械新村、白云东苑、清潭五村、广成小区、勤业六村及新区燕兴新村通公交车问题；(5)实施“放心肉菜”工程，凌家塘、苏浙皖边界农副产品批发市场配备检测中心，100家零售农贸市场配备检测室或检测设备，为市民饮食消费提供安全保障；(6)深入开展市河整治，对龙游河、南市河的部分河段进行整治，实施横塘浜北环新村段护岸和绿化工程；(7)发展社区卫生服务，所有街道建立社区卫生服务中心，80%的社区居委会建立卫生工作站；(8)改善城乡居民用水质量，解决新北区三井、龙虎塘2个乡镇，钟楼区新闸、北港2个乡镇，以及武进区湖塘等22个镇饮用长江水问题，受益人口近20万人；(9)改造县乡公路114.5公里，到2004年底实现金坛、溧阳市区至各乡镇通二级以上公路，乡镇之间通三级以上公路；(10)对8个渡口实施撤渡建桥，明年底前完成全市所有渡口的撤渡建桥工作。

　　各位代表，新的时代孕育着新的机遇，新的时代充满着新的挑战。让我们在省委、省政府和市委的领导下，以邓小平理论和“三个代表”重要思想为指导，全面贯彻落实党的十六大精神，以只争朝夕、奋发有为的精神状态，艰苦创业、追求卓越的坚定信念，脚踏实地、求真务实的工作作风，团结拼搏，开拓进取，全面推进我市改革开放和现代化建设的宏伟大业，为共同创造我们的幸福生活和美好未来，为加快富民强市、率先基本实现现代化而努力奋斗!
